# Does knowing the influenza epidemic threshold has been reached influence the performance of influenza case definitions?

**DOI:** 10.1371/journal.pone.0270740

**Published:** 2022-07-01

**Authors:** Núria Soldevila, Diana Toledo, Ana Martínez, Pere Godoy, Núria Torner, Cristina Rius, Mireia Jané, Angela Domínguez, the PIDIRAC Sentinel Surveillance Program of Catalonia

**Affiliations:** 1 CIBER Epidemiología y Salud Pública (CIBERESP), Madrid, Spain; 2 Departament de Medicina, Universitat de Barcelona, Barcelona, Spain; 3 Agència de Salut Pública de Catalunya, Generalitat de Catalunya, Barcelona, Spain; 4 Agència de Salut Pública de Barcelona, Barcelona, Spain; ISI Foundation: Fondazione ISI - Istituto per l’lnterscambio Scientifico, ITALY

## Abstract

**Background:**

Disease surveillance using adequate case definitions is very important. The objective of the study was to compare the performance of influenza case definitions and influenza symptoms in the first two epidemic weeks with respect to other epidemic weeks.

**Methods:**

We analysed cases of acute respiratory infection detected by the network of sentinel primary care physicians of Catalonia for 10 seasons. We calculated the diagnostic odds ratio (DOR) and 95% confidence intervals (CI) for the first two epidemic weeks and for other epidemic weeks.

**Results:**

A total of 4,338 samples were collected in the epidemic weeks, of which 2,446 (56.4%) were positive for influenza. The most predictive case definition for laboratory-confirmed influenza was the WHO case definition for influenza-like illness (ILI) in the first two epidemic weeks (DOR 2.10; 95% CI 1.57–2.81) and in other epidemic weeks (DOR 2.31; 95% CI 1.96–2.72). The most predictive symptom was fever. After knowing that epidemic threshold had been reached, the DOR of the ILI WHO case definition in children aged <5 years and cough and fever in this group increased (190%, 170% and 213%, respectively).

**Conclusions:**

During influenza epidemics, differences in the performance of the case definition and the discriminative ability of symptoms were found according to whether it was known that the epidemic threshold had been reached or not. This suggests that sentinel physicians are stricter in selecting samples to send to the laboratory from patients who present symptoms more specific to influenza after rather than before an influenza epidemic has been declared.

## Introduction

Influenza epidemics are associated with excess morbidity and mortality each season [1]. Worldwide, annual influenza epidemics are estimated to result in about 3 to 5 million cases of severe illness and about 290,000 to 650,000 respiratory deaths [2].

The start, peak, duration, and intensity of individual seasons vary substantially from year to year and reflect the interplay of several factors, including the extent of the antigenic variation of the virus, virulence and transmissibility, the extent of immunity in the population, and the specific population groups affected [3]. Thus, influenza surveillance is very important from a public health perspective.

To manage influenza epidemics appropriately, it is important to inform physicians, clinical staff and the general population about the onset of the influenza epidemic. However, and despite occurring yearly, the onset of the influenza epidemic is unpredictable. Reliable and timely information on current influenza activity are of the utmost interest to health services and health-related decision makers [4].

In Catalonia, the PIDIRAC (Pla d’Informació de les Infeccions Respiratòries Agudes a Catalunya) was responsible for collecting and reporting data on influenza activity in each season during the study period. This surveillance mechanism relies on a network of sentinel physicians who collect samples of influenza-like illness (ILI) or acute respiratory infection (ARI) syndromes following ECDC case definitions from patients attended by primary care physicians from week 40 to week 20 each season. Announcement of the onset of influenza epidemic activity is deployed through direct contact with physicians involved in the surveillance tasks and by the weekly report published on the webs related to the Department of Health of Catalonia. Also through a press conference the health authorities inform the onset of the epidemic for release to mass media. Even if consultations are not delayed, data is available, at best, after a one-week lag. This means that between the real onset of seasonal epidemic activity and the official alert, several weeks can elapse [4].

Case definitions are an important part of standardized systems for public health surveillance. The World Health Organization (WHO) defines influenza-like illness as an acute respiratory illness with a measured temperature of ≥38°C and cough, with onset within the last 10 days [5]. The European Center for Disease Prevention and Control (ECDC) defines ILI as the sudden onset of symptoms and at least one of four systemic symptoms: fever or feverishness, malaise, headache, myalgia, and at least one of three respiratory symptoms: cough, sore throat, and shortness of breath and defines ARI as the sudden onset of symptoms and at least one of four respiratory symptoms: cough, sore throat, shortness of breath or coryza [6].

According to the PIDIRAC system, a week is considered epidemic when it exceeds the established epidemic threshold which, in each season, is based on incidence data from epidemic periods in preceding seasons [7].

Knowing that the influenza epidemic has begun might influence the decision of primary care sentinel physicians to collect samples from patients [8].

The objective of this study was to compare the performance of the ECDC and WHO influenza case definitions and influenza symptoms in the first two epidemic weeks with respect to other epidemic weeks.

## Methods

### Study design

We made a retrospective study of the clinical and epidemiological characteristics of cases of ARI detected by the primary care influenza sentinel physicians’ network during ten influenza seasons (2008–2009 to 2017–2018) in Catalonia, a Spanish region with 7.6 million inhabitants.

Each sentinel physician collected weekly nasopharyngeal swabs in up to two individuals who presented symptoms compatible with ARI or ILI and sent them to the network’s laboratory for determination of respiratory viruses (influenza viruses A-C, syncytial respiratory virus, parainfluenza viruses 1–4, adenovirus, coronavirus, rhinovirus, metapneumovirus and bocavirus) by real-time reverse transcriptase-polymerase chain reaction (RT-PCR). Individuals whose clinical samples showed coinfection were excluded.

### Data collection

For each individual, we collected the following variables: recorded fever >37.8°C, cough, malaise, headache, myalgia, sore throat, shortness of breath, sudden onset of symptoms, coryza, age, sex, comorbidities (chronic cardiovascular disease, pulmonary disease including asthma, liver disease, renal disease, metabolic disorders including diabetes mellitus, obesity [defined as a body mass index (BMI) >40 Kg/m^2^ for adults and a BMI at or above the 95th percentile for children and teens of the same age and sex] and immunodeficiency) and microbiological laboratory results.

The ECDC and WHO case definitions for ILI were analysed. A temperature of ≥37.8°C was used to assess the WHO ILI case definition, as this is the variable recorded on the form used by sentinel physicians. Influenza epidemic weeks were defined each season according to the data provided by the PIDIRAC [7].

### Statistical analysis

For case definitions and influenza symptoms we calculated the sensitivity (Se), specificity (Sp), positive predictive value (PPV), positive likelihood ratio (LR), negative LR, diagnostic odds ratio (DOR) and their 95% confidence intervals (CI), comparing cases in which influenza virus was detected with cases positives for other respiratory viruses or negatives for all viruses. Sensitivity was defined as the proportion of patients identified by case definitions among those with a positive result for influenza. Specificity was defined as the proportion of subjects not identified by case definitions among those with a negative result for influenza. PPV was defined as the proportion of confirmed influenza patients among those who met the case definition. These calculations were made for the first two epidemic weeks (before it was known the epidemic threshold had been reached) and for other epidemic weeks globally and by age groups, sex and presence of comorbidities.

DOR was defined as the positive LR/negative LR. The DORs were predictors of laboratory-confirmed influenza when they were greater than 1 and their 95% CI did not contain the value 1.

Analyses were also performed separating for the first epidemic week and the first three epidemic weeks.

The analysis was performed using the SPSS version 25 statistical package and R version 3.5.0 statistical software.

### Ethical considerations

All data were collected as part of routine public health surveillance activities according to the legal mandate of the Health Department of Catalonia, which is officially authorized to receive, treat and temporarily store personal data in cases of infectious diseases [9]. All data were fully anonymized. All study activities formed part of public health surveillance and were exempt from institutional board review and did not require informed consent.

## Results

**Fig 1:**
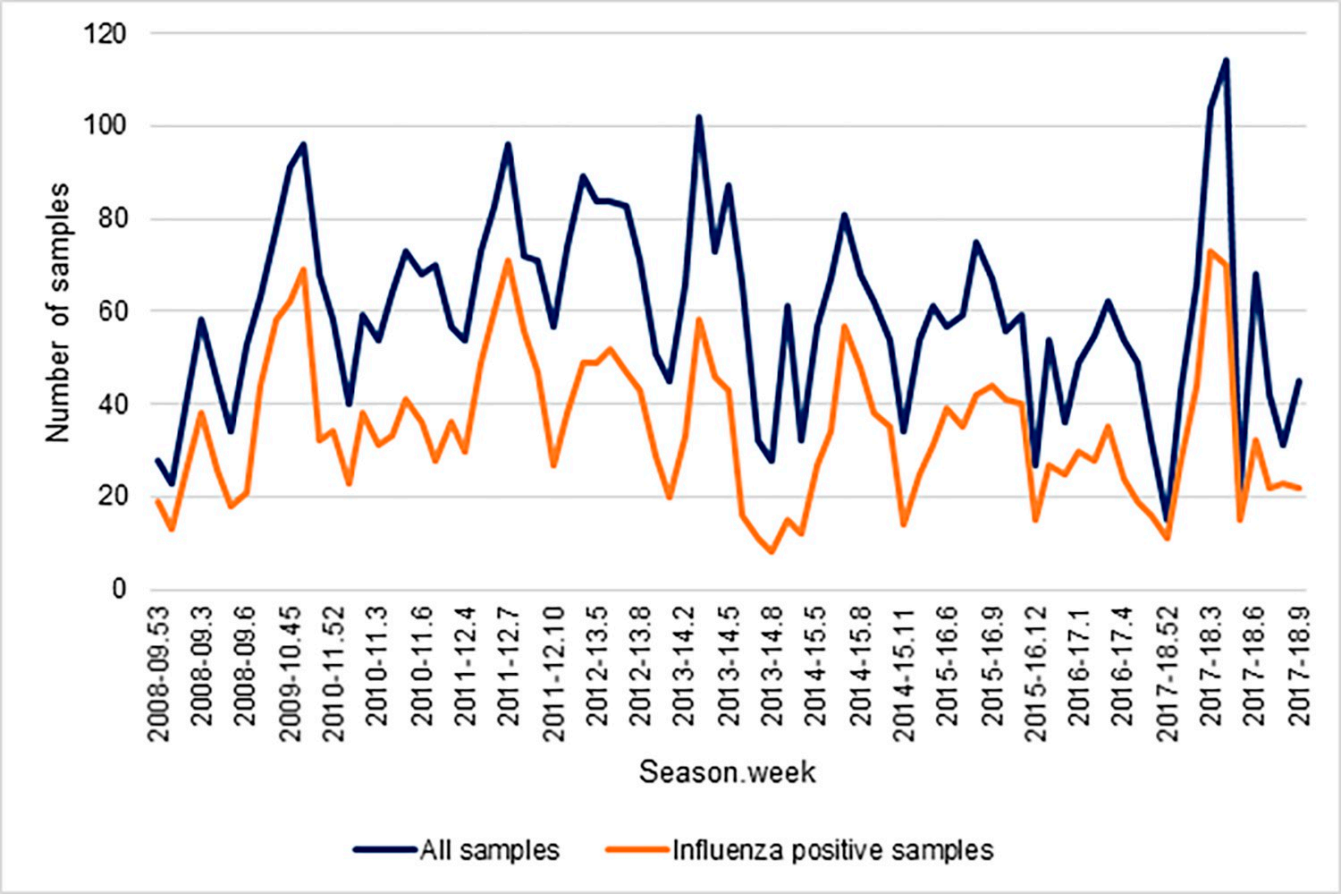
Number of samples collected and positive samples for influenza by season and week.

During the ten influenza seasons studied, 4,637 samples were collected, of which 299 were excluded due to coinfection. Therefore 4,338 samples were analysed, of which 2,446 (56.4%) were positive for influenza. Number of samples collected and positive samples for influenza by season and week was show in Fig 1. The most frequent clinical manifestations in the first two epidemic weeks and other epidemic weeks were fever (89.6% and 92.0%, respectively) and cough (84.1% and 82.8%, respectively) (Table 1).

**Table 1:** Influenza-positive and negative patients included in the study. Influenza sentinel surveillance system, Catalonia, 2008–2018.

	All cases		Positive for influenza		Negative for influenza	
	**n**	**%**	**n**	**%**	**n**	**%**
**FIRST TWO EPIDEMIC WEEKS**	1000		520		480	
**Case definition**						
ECDC ILI	570	57.0	324	62.3	246	51.2
WHO ILI	747	74.7	423	81.3	324	67.5
**Clinical symptoms**						
Fever	896	89.6	493	94.8	403	84.0
Cough	841	84.1	448	86.2	393	81.9
Malaise	691	69.1	350	67.3	341	71.0
Headache	448	44.8	259	49.8	189	39.4
Myalgia	540	54.0	299	57.5	241	50.2
Sore throat	527	52.7	272	52.3	255	53.1
Shortness of breath	57	5.7	17	3.3	40	8.3
Sudden onset of symptoms	630	63.0	350	67.3	280	58.3
**Age**						
0–4 years	235	23.5	93	17.9	142	29.6
5–14 years	273	27.3	195	37.5	78	16.3
15–64 years	423	42.3	210	40.4	213	44.4
≥65 years	69	6.9	22	4.2	47	9.8
**Comorbidities**						
Yes	98	9.8	35	6.7	63	13.1
No	902	90.2	485	93.3	417	86.9
**OTHER EPIDEMIC WEEKS**	3338		1926		1412	
**Case definition**						
ECDC ILI	1796	53.8	1097	57.0	699	49.5
WHO ILI	2541	76.1	1591	82.6	950	67.3
**Clinical symptoms**						
Fever	3071	92.0	1832	95.1	1239	87.7
Cough	2765	82.8	1670	86.7	1095	77.5
Malaise	2378	71.2	1411	73.3	967	68.5
Headache	1603	48.0	986	51.2	617	43.7
Myalgia	1807	54.1	1083	56.2	724	51.3
Sore throat	1693	50.7	981	50.9	712	50.4
Shortness of breath	172	5.2	73	3.8	99	7.0
Sudden onset of symptoms	2015	60.4	1198	62.2	817	57.9
**Age**						
0–4 years	689	20.6	359	18.6	330	23.4
5–14 years	954	28.6	649	33.7	305	21.6
15–64 years	1489	44.6	815	42.3	674	47.7
≥65 years	206	6.2	103	5.3	103	7.3
**Comorbidities**						
Yes	352	10.5	199	10.3	153	10.8
No	2986	89.5	1727	89.7	1259	89.2

The case definition that was the most predictive of laboratory-confirmed influenza was the WHO case definition for ILI, both in the first two epidemic weeks (DOR 2.10; 95% CI 1.57–2.81) and in other epidemic weeks (DOR 2.31; 95% CI 1.96–2.72). Clinical manifestations that were significant positive predictors of laboratory-confirmed influenza were fever, cough, headache, myalgia, and sudden onset of symptoms. Fever had the highest DOR in the first two epidemic weeks (DOR 3.49; 95% CI 2.21–5.51) and in other epidemic weeks (DOR 2.72; 95% CI 2.10–3.53) (Table 2).

**Table 2:** Sensitivity, specificity, positive predictive value, likelihood ratios, DOR of case definitions and clinical symptoms for the first two epidemic weeks and for other epidemic weeks. Influenza sentinel surveillance system, Catalonia, 2008–2018.

	First two epidemic weeks							Other epidemic weeks					
Case definition	Se (%)	Sp (%)	PPV (%)	Positive LR (95% CI)	Negative LR (95% CI)	DOR (95% CI)	Se (%)		Sp (%)	PPV (%)	Positive LR (95% CI)	Negative LR (95% CI)	DOR (95% CI)
ECDC ILI	62 (58–66)	49 (44–53)	57 (53–61)	1.22 (1.09–1.36)	0.77 (0.67–0.89)	1.57 (1.22–2.02)	57 (55–59)		50 (48–53)	61 (59–63)	1.15 (1.08–1.23)	0.85 (0.79–0.92)	1.35 (1.18–1.55)
WHO ILI	81 (78–85)	32 (28–37)	57 (53–60)	1.21 (1.12–1.30)	0.57 (0.46–0.72)	2.10 (1.57–2.81)	83 (81–84)		53 (30–35)	63 (61–64)	1.23 (1.18–1.28)	0.53 (0.47–0.60)	2.31 (1.96–2.72)
Fever	95 (93–97)	16 (13–20)	55 (52–58)	1.13 (1.08–1.18)	0.32 (0.21–0.49)	3.49 (2.21–5.51)	95 (94–96)		12 (11–14)	60 (58–61)	1.08 (1.06–1.11)	0.40 (0.31–0.51)	2.72 (2.10–3.53)
Cough	86 (83–89)	18 (15–22)	53 (50–57)	1.05 (1.00–1.11)	0.76 (0.57–1.02)	1.38 (0.98–1.94)	87 (85–88)		22 (20–25)	60 (59–62)	1.12 (1.08–1.16)	0.59 (0.51–0.69)	1.89 (1.57–2.26)
Malaise	67 (63–71)	29 (25–33)	51 (47–54)	0.95 (0.87–1.03)	1.13 (0.94–1.36)	0.84 (0.64–1.10)	73 (71–75)		32 (29–34)	59 (57–61)	1.07 (1.02–1.12)	0.85 (0.76–0.94)	1.26 (1.08–1.47)
Headache	50 (45–54)	61 (56–65)	58 (53–62)	1.26 (1.10–1.46)	0.83 (0.74–0.93)	1.53 (1.19–1.96)	51 (49–53)		56 (54–59)	62 (59–64)	1.17 (1.09–1.26)	0.87 (0.81–0.92)	1.35 (1.18–1.55)
Myalgia	57 (53–62)	50 (45–54)	55 (51–60)	1.15 (1.02–1.29)	0.85 (0.75–0.98)	1.34 (1.04–1.72)	56 (54–58)		49 (46–51)	60 (58–62)	1.10 (1.03–1.17)	0.90 (0.83–0.97)	1.22 (1.06–1.40)
Sore throat	52 (48–57)	47 (42–51)	52 (47–56)	0.98 (0.88–1.11)	1.02 (0.89–1.16)	0.97 (0.75–1.24)	51 (49–53)		50 (47–52)	58 (56–60)	1.01 (0.94–1.08)	0.99 (0.94–1.08)	1.02 (0.89–1.17)
Shortness of breath	3 (2–5)	92 (89–94)	30 (18–43)	0.39 (0.23–0.68)	1.06 (1.02–1.09)	0.37 (0.21–0.66)	4 (3–5)		93 (92–94)	42 (35–50)	0.54 (0.40–0.73)	1.03 (1.02–1.05)	0.52 (0.38–0.71)
Sudden onset of symptoms	67 (63–71)	42 (37–46)	56 (52–59)	1.15 (1.05–1.27)	0.78 (0.67–0.92)	1.47 (1.14–1.90)	62 (60–64)		42 (40–45)	59 (57–62)	1.08 (1.02–1.14)	0.90 (0.82–0.98)	1.20 (1.04–1.38)

DOR: Diagnostic odds ratio; Se: Sensitivity, Sp: specificity, PPV: positive predictive value

**Fig 2:**
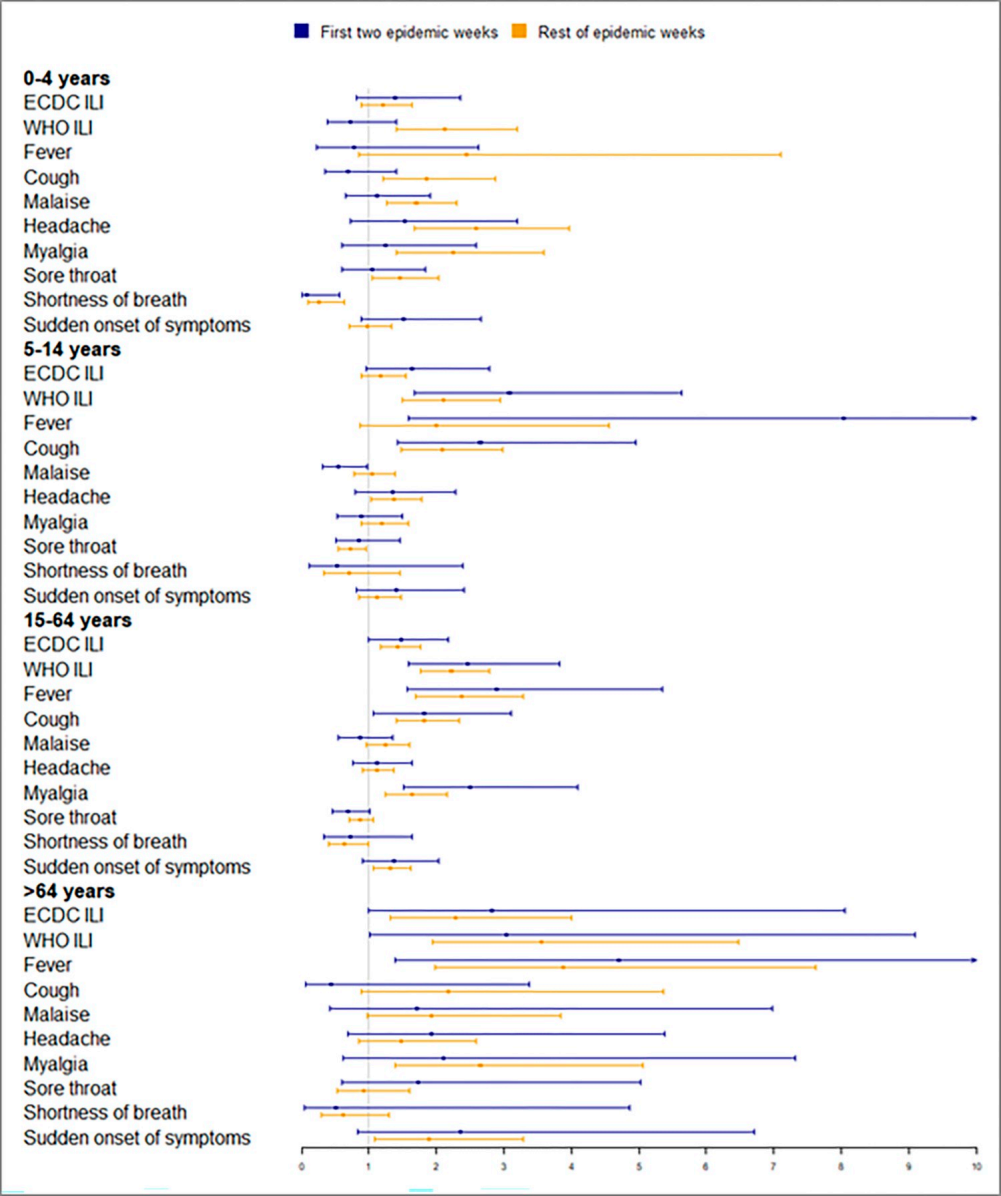
DOR of clinical manifestations for the first two epidemic weeks and rest of epidemic weeks by age group.

**Fig 3:**
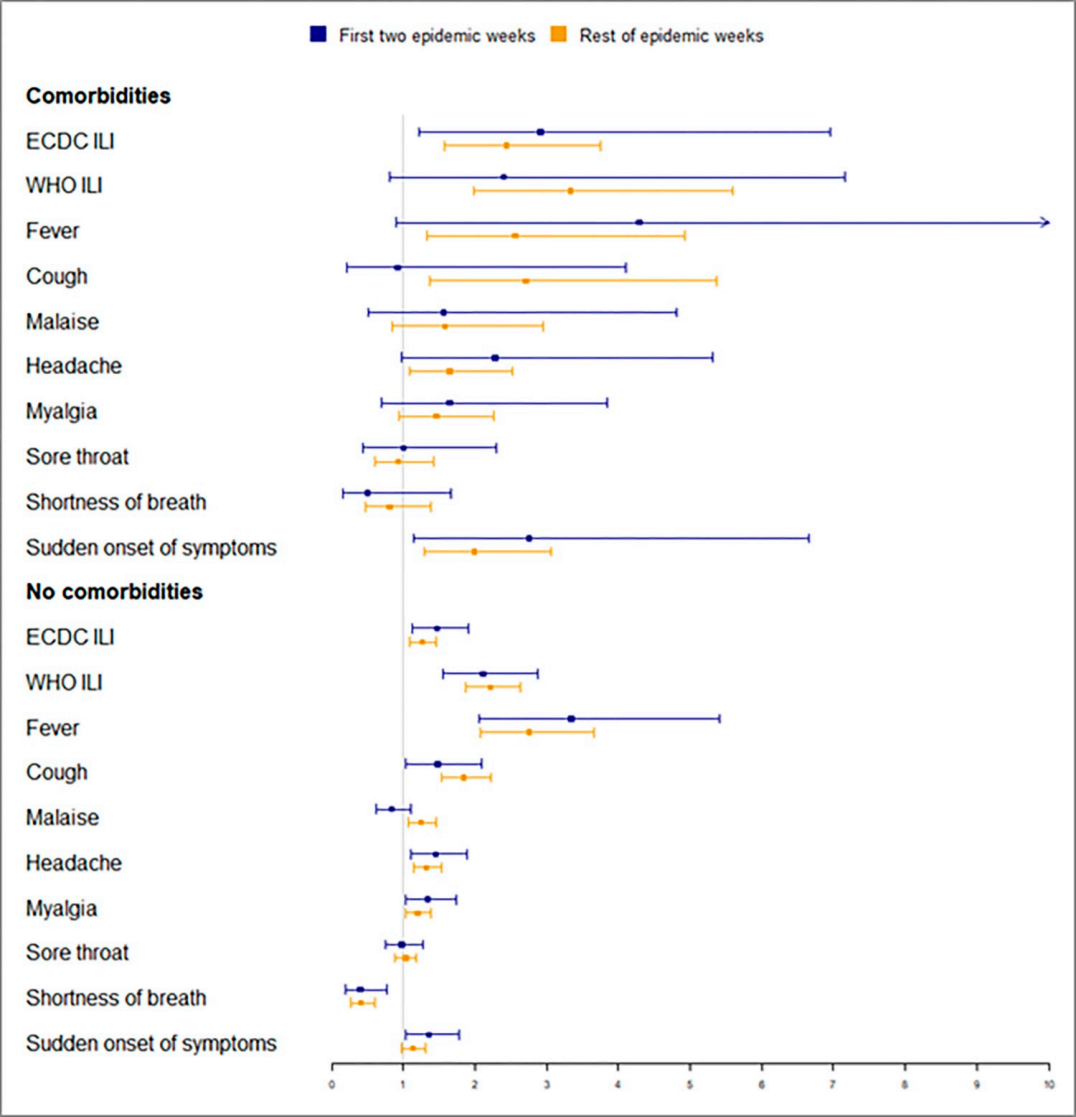
DOR of clinical manifestations for the first two epidemic weeks and rest of epidemic weeks by comorbidities.

The performance of the case definitions and specific clinical manifestations by age group and comorbidities for the first two epidemic weeks is shown in Fig 2 and S1 Table and for other epidemic weeks in Fig 3 and S2 Table. The 0–4 age group was the group where the most differences were observed between the two periods. After knowing that the epidemic threshold had been reached, the DOR of the WHO ILI case definition in children aged <5 years, the DOR of cough and the DOR of fever in children aged <5 years increased (190%, 170% and 213%, respectively), as did the DOR of malaise in all patients (50%), in those aged 5–14 years (44%) and in patients without comorbidities (49%).

The performances of influenza case definitions and influenza symptoms in the first epidemic weeks and in the first three epidemic weeks are show in S3 and S4 Tables. The case definition that was the most predictive of laboratory-confirmed influenza was the WHO case definition for ILI and fever had the highest DOR both in the first epidemic weeks, in the first three weeks and in other epidemic weeks.

## Discussion

The case definition that was the best predictor of laboratory-confirmed influenza was the WHO case definition for ILI, both in the first two epidemic weeks (DOR 2.10; 95% CI 1.57–2.81) and in other epidemic weeks (DOR 2.31; 95% CI 1.96–2.72). After knowing that the epidemic threshold had been reached, the DOR of the ILI WHO case definition in patients aged <5 years increased by 190%, but no significant differences were observed in all patients and in the other age groups.

In a German study in the 2013 influenza season, the performance of the WHO case definition was 2.06, similar to our results [10]. In a French study carried out in 2009–2014, the performance of the WHO case definition was better than that of the ECDC case definition (2.40 vs 1.74) [11]. A study carried out in Singapore in 2005–2009 also found that the performance of the WHO case definition was better than the ECDC case definition (13.5 vs 9.7) [12]. In a study carried out in Senegal during 2013 and 2016, the WHO and ECDC definitions showed a similar performance, including by age group; the authors suggest the WHO ILI definition could be chosen for surveillance purposes due to its simplicity [13].

Fever had the best performance in the first two epidemic weeks (DOR 3.49; 95% CI 2.21–5.51) and in other epidemic weeks (DOR 2.72; 95% CI 2.10–3.53); this performance was superior for both WHO and ECDC case definitions. After knowing that the epidemic threshold had been reached, the performance of fever in patients aged <5 years increased by 213%. A Pakistan study in 2009–2010 found that fever had the best performance, both in children and adults [14]. However, a study by Chughtai et al. found that fever was less common in adults with confirmed viral respiratory infections, including influenza, than in children [15].

Cough was the symptom with the second-best performance in other epidemic weeks (DOR 1.89; 95% CI 1.57–2.26). After knowing that the epidemic threshold had been reached, the performance of cough in patients aged <5 years increased by 170% (DOR 1.86; 95% CI 1.21–2.87). In the aforementioned Senegalese study, cough was significantly associated with influenza in children aged <5 and ≥5 years (OR 2.21 and 2.70, respectively) [13]. A study carried out in Illinois in 2009–2011 found that the best predictors of laboratory-confirmed influenza were cough followed by fever (DOR 6.39 and 4.7, respectively) [16]. Woolpert et al., in a USA study in 2007–2008, also reported cough (DOR 47.99) followed by fever (DOR 3.84) to be the best predictors [17].

A Canadian study in 1998–1999 found that cough and fever had an 86.6% positive predicted value [18]. Other authors have found that cough and fever are the strongest clinical predictors of influenza, regardless of age group, suggesting that a simplified case definition of fever and cough may be suitable in all ages [12, 16].

Headache and myalgia in both the first two epidemic weeks and in the other epidemic weeks and malaise in the other epidemic weeks were also predictors of influenza. Other authors found that rhinorrhoea, headache and myalgia were symptoms reported as significant predictors of influenza infection that could be used by physicians when deciding treatment [11, 16, 19].

The study by Woolpert et al. found that patterns of influenza predictors differed across age groups and that no combination of clinical and demographic predictors served as a reliable diagnostic case definition in the population studied [17], but suggested that the case definition is useful if it is accompanied by rapid tests in all patients [17]. In addition, knowledge of the causative agent might have a considerable influence on case management, especially in children, where the specific diagnosis of a viral pathogen may avoid overtreatment and antibiotics [10].

An Ukrainian study carried out during the early weeks of the 2015–2016 influenza season found that the early and steep increase in severe cases and deaths was associated with intensive media attention on the influenza season and this may have led to increased awareness of influenza infection among physicians and the public [20]. Some authors also state that, as the levels of influenza activity increase, the accuracy of case definitions tends to improve, which is consistent with the fact that the clinical identification of influenza is improved when physicians are aware of influenza virus circulation in their geographic area [8, 21]. Therefore, it is important to report an increase in influenza activity using the surveillance system. In addition, as found by Won et al., the onset of the flu season may be detected and consistently anticipated, regardless of the amplitude of the epidemic, which is an obvious advantage for health decision makers [4].

The case definition used for influenza surveillance may be helpful during the COVID-19 pandemic as timely use of antiviral influenza drugs together with booster influenza vaccination can reduce the social and economic burden of influenza [22].

In the same way that we wanted to ascertain whether the performance of case definitions and clinical presentation varies according to the time of the epidemic, several authors have studied the usefulness of the available information with the aim of better controlling seasonal epidemics. Moss et al. found that forecasting methods can quantify the impact of delays in data availability and variable reporting practices on the accuracy of the current epidemic assessment [23]. Kandula et al. suggested that supplementing surveillance data with proxy estimates improves the quality of forecasting and that transient errors degrade the performance [24].

Our study had strengths and limitations. The main strength is that we only included patients with a laboratory‐confirmed diagnosis of influenza, thus eliminating verification bias [25], and that all physicians were working in sentinel primary healthcare centres using the same criteria for clinical sampling. Another strength is that other possible aetiologies of acute respiratory infections were studied in all samples, excluding those with coinfection [26].

A limitation is that, in our sentinel surveillance network, fever was predefined as a temperature >37.8°C, but the specific temperature was not reported for the cases included in the study. However, the impact of this limitation may be minimal, as other factors, such as individual and daily variations, the site of measurement and the natural trend for physicians to round temperatures up or down, can also influence the measured temperature [11].

In conclusion, during influenza epidemics, differences in the performance of the case definition and the discriminative ability of symptoms were observed according to whether it was known that the epidemic threshold had been reached or not, especially in children aged 0–4 years. Our results suggest that sentinel physicians are stricter in selecting samples to send to the laboratory from patients who present symptoms more specific to influenza after rather than before the influenza epidemic is declared.

## Supporting information

S1 Table DOR of clinical manifestations for the first two epidemic weeks, stratified by age group and comorbidities. (DOCX)

S2 Table DOR of clinical manifestations for other epidemic weeks, stratified by age group and comorbidities. (DOCX)

S3 Table Sensitivity, specificity, positive predictive value, likelihood ratios, DOR of case definitions and clinical symptoms for the first epidemic weeks and for other epidemic weeks. Influenza sentinel surveillance system, Catalonia, 2008–2018.
(DOCX)

S4 Table Sensitivity, specificity, positive predictive value, likelihood ratios, DOR of case definitions and clinical symptoms for the first three epidemic weeks and for other epidemic weeks. Influenza sentinel surveillance system, Catalonia, 2008–2018.
(DOCX)

S1 Data (XLSX)

## References

[1] Treanor JJ. Influenza Viruses, including avian influenza and swine influenza. In: Bennett JE, Dolin R, Blaser MJ, editors. Principles and Practice of Infectious Diseases. 9th ed. Philadelphia: Elsevier; 2020. pp. 2143–2168.

[2] WHO. Influenza (Seasonal) [cited 26 June 2021]. Available from: https://www.who.int/en/news-room/fact-sheets/detail/influenza-(seasonal)

[3] Bresee JS, Fry A, Sambhara S, Cox NJ. Inactivated influenza vaccines. In: Plotkin SA, Orenstein WA, Offit PA, Edwards KM, editors. Vaccines. 7th ed. Philadelphia: Saunders; 2008. pp. 456–488.

[4] Won M, Marques-Pita M, Louro C, Gonçalves-Sá J. Early and real-time detection of seasonal influenza onset. PLoS Comput Biol. 2017;13: e1005330. doi: 10.1371/journal.pcbi.1005330 28158192 PMC5291378

[5] World Health Organization. WHO Surveillance Case Definitions for ILI and SARI. Geneva: WHO Press; 2014.

[6] Commission European. Commission implementing decisions (EU) 2018/945 of 22 June 2018 on the communicable diseases and related special health issues to be covered by epidemiological surveillance as well as relevant case definitions. Official Journal of the European Union. 2018;170: 1–74.

[7] PIDIRAC. Pla d’informació de les infeccions respiratòries agudes a Catalunya (PIDIRAC) 2016–2017. [cited 28 June 2021]. Available from: https://scientiasalut.gencat.cat/bitstream/handle/11351/3385/pla_informacio_infeccions_respiratories_agudes_catalunya_pidirac_2016_2017.pdf?sequence=1&isAllowed=y

[8] Montalto NJ. An office-based approach to influenza: clinical diagnosis and laboratory testing. Am Fam Physician. 2003;67: 111–118. 12537174

[9] Decret 2013/2015, de 15 de setembre, pel qual es crea la Xarxa de Vigilància Epidemiològica i es regulen els sistemes de notificació de malalties de declaració obligatòria i els brots epidèmics. DOGC. 2015;6958: 1–19.

[10] Campe H, Heinzinger S, Hartberger C, Sing A. Clinical symptoms cannot predict influenza infection during the 2013 influenza season in Bavaria, Germany. Epidemiol Infect. 2016;144: 1045–1051. doi: 10.1017/S0950268815002228 26388141

[11] Casalegno J, Eibach D, Valette M, Enouf V, Daviaud I, Behillil S, et al. Performance of influenza case definitions for influenza community surveillance: based on the French influenza surveillance network GROC, 2009–2017. Euro Surveill. 2017;22: pii = 30504. doi: 10.2807/1560-7917.ES.2017.22.14.30504 28422004 PMC5388124

[12] Jiang L, Lee VJ, Lim WY, Chen MI, Chen Y, Tan L, et al. Performance of case definitions for influenza surveillance. Euro Surveill. 2015;20: pii = 21145. doi: 10.2807/1560-7917.es2015.20.22.21145 26062645

[13] Barry MA, Arinal F, Talla C, Hedible BG, Sarr FD, Ba IO, et al. Performance of case definitions and clinical predictors for influenza surveillance among patients followed in a rural cohort in Senegal. BMC Infectious Diseases. 2021;21: 31. doi: 10.1186/s12879-020-05724-x 33413174 PMC7790019

[14] Nisar N, Aamir UB, Badar N, Mehmood MR, Alam MM, Kazi BM, et al. Prediction of Clinical Factors Associated with Pandemic Influenza A (H1N1) 2009 in Pakistan. PLoS ONE. 2014;9: e89178. doi: 10.1371/journal.pone.0089178 24586575 PMC3933350

[15] Chughtai AA, Wang Q, Dung TC, Macintyre CR. The presence of fever in adults with influenza and other viral respiratory infections. Epidemiol Infect. 2017;145: 148–155. doi: 10.1017/S0950268816002181 27691995 PMC5197931

[16] Shah SC, Rumoro DP, Hallock MM, Trenholme GM, Gibbs GS, Silva JC, et al. Clinical predictors for laboratory‐confirmed influenza infections: Exploring case definitions for influenza‐like illness. Infect Control Hosp Epidemiol. 2015;36: 241–248. doi: 10.1017/ice.2014.64 25695163

[17] Woolpert T, Brodine S, Lemus H, Waalen J, Blair P, Faix D. Determination of clinical and demographic predictors of laboratory-confirmed influenza with subtype analysis. BMC Infectious Diseases. 2012;12: 129. doi: 10.1186/1471-2334-12-129 22676850 PMC3407722

[18] Boivin G, Hardy I, Tellier G, Maziade J. Predicting influenza infections during epidemics with use of a clinical case definition. Clinl Infect Dis. 2000;31: 1166–1169. doi: 10.1086/317425 11073747

[19] Monamele CG, Kengne-Nde C, Munshili Njifon HL, Njankouo MR, Kenmoe S, Njouom R. Clinical signs predictive of influenza virus infection in Cameroon. PLoS ONE. 2020;15: e0236267. doi: 10.1371/journal.pone.0236267 32701976 PMC7377385

[20] Newit S, Mironenko A, Holubka O, Zaika O, Gubar O, Jalava K, et al. Rapid risk assessment during the early weeks of the 2015–2016 influenza season in Ukraine. Influenza Other Respi Viruses. 2018;12: 241–249. 10.1111/irv.12526 PMC5820420 29152877

[21] Yang TU, Cheong HJ, Song JY, Lee JS, Wie SH, Kim YK, et al. Age- and influenza activity-stratified case definitions of influenza-like illness: experience from hospital-based influenza surveillance in South Korea. PLoS ONE. 2014;9: e84873. doi: 10.1371/journal.pone.0084873 24475034 PMC3901651

[22] Murillo-Zamora E, Hernández-Suárez C. Performance of the case definition of suspected influenza before and during the COVID-19 pandemic. Rev Clin Esp. 2021; Online ahead of print. doi: 10.1016/j.rce.2020.09.001 34839891 PMC7997690

[23] Moss R, Fielding JE, Franklin LJ, Stephens N, McVernon J, Dawson P, et al. Epidemic forecasts as a tool for public health: interpretation and (re)calibration. Aust N Z J Public Health. 2018;42: 69–76. doi: 10.1111/1753-6405.12750 29281169

[24] Kandula S, Yamana T, Pei S, Yang W, Morita H, Shaman J. Evaluation of mechanistic and statistical methods in forecasting influenza-like illness. J R Soc Interface. 2018;15: 20180174. doi: 10.1098/rsif.2018.0174 30045889 PMC6073642

[25] Call SA, Vollenweider MA, Hornung CA, Simel DL, McKinney QP. Does this patient have influenza? JAMA. 2005;293: 987–997. doi: 10.1001/jama.293.8.987 15728170

[26] Kim JM, Jung HD, Cheong HM, Lee A, Lee NH, Chu H, et al. Nation‐wide surveillance of human acute respiratory virus infection between 2013 and 2015 in Korea. J Med Virol. 2018;90: 1177–1183. doi: 10.1002/jmv.25069 29488229 PMC7166751

