# Supplementary material for: Does knowing the influenza epidemic threshold has been reached influence the performance of influenza case definitions?
Source: PLoS One. 2022 Jul 1;17(7):e0270740. doi: 10.1371/journal.pone.0270740 (PMC9249166; doi:10.1371/journal.pone.0270740)
Supplement: S1 Table — (DOCX) [file pone.0270740.s001.docx]

**S1 Table.** DOR of clinical manifestations for the first two epidemic weeks, stratified by age group and comorbidities.

|  | **0-4 years** | **5-14 years** | **15-64 years** | **≥65 years** | **Comorbidities** | **No comorbidities** |
| --- | --- | --- | --- | --- | --- | --- |
|  | **DOR**  **(95% CI)** | **DOR**  **(95% CI)** | **DOR**  **(95% CI)** | **DOR**  **(95% CI)** | **DOR**  **(95% CI)** | **DOR**  **(95% CI)** |
| **Case definition** |  |  |  |  |  |  |
| ECDC ELI | 1.39 (0.82-2.36) | 1.63 (0.96-2.78) | 1.47 (1.00-2.17) | 2.82 (0.99-8.05) | 2.91 (1.22-6.95) | 1.47 (1.12-1.91) |
| WHO ILI | 0.73 (0.38-1.40) | 3.08 (1.68-5.64) | 2.46 (1.58-3.82) | 3.03 (1.01-9.10) | 2.40 (0.80-7.16) | 2.11 (1.56-2.87) |
| **Clinical symptoms** |  |  |  |  |  |  |
| Fever | 0.78 (0.23-2.62) | 8.04 (1.59-40.76) | 2.89 (1.57-5.34) | 4.70 (1.38-15.99) | 4.29 (0.91-20.26) | 3.34 (2.06-5.40) |
| Cough | 0.69 (0.34-1.41) | 2.65 (1.42-4.95) | 1.82 (1.06-3.11) | 0.44 (0.06-3.38) | 0.92 (0.21-4.10) | 1.48 (1.04-2.10) |
| Malaise | 1.12 (0.66-1.91) | 0.55 (0.31-0.98) | 0.86 (0.54-1.35) | 1.71 (0.42-6.97) | 1.56 (0.51-4.81) | 0.84 (0.63-1.11) |
| Headache | 1.53 (0.73-3.20) | 1.35 (0.80-2.28) | 1.11 (0.76-1.63) | 1.93 (0.69-5.39) | 2.28 (0.97-5.32) | 1.45 (1.11-1.89) |
| Myalgia | 1.25 (0.60-2.59) | 0.88 (0.52-1.50) | 2.49 (1.51-4.10) | 2.11 (0.61-7.32) | 1.65 (0.69-3.85) | 1.34 (1.03-1.74) |
| Sore throat | 1.04 (0.59-1.84) | 0.85 (0.50-1.46) | 0.68 (0.46-1.01) | 1.73 (0.60-5.02) | 1.00 (0.43-2.30) | 0.98 (0.75-1.27) |
| Shortness of breath | 0.07 (0.01-0.57) | 0.52 (0.11-2.39) | 0.73 (0.33-1.63) | 0.51 (0.05-4.87) | 0.50 (0.15-1.66) | 0.40 (0.20-0.78) |
| Sudden onset of symptoms | 1.52 (0.88-2.65) | 1.40 (0.82-2.40) | 1.37 (0.91-2.04) | 2.36 (0.83-6.71) | 2.75 (1.14-6.66) | 1.36 (1.03-1.78) |

DOR: Diagnostic Odds Ratio
