# Supplementary material for: Does knowing the influenza epidemic threshold has been reached influence the performance of influenza case definitions?
Source: PLoS One. 2022 Jul 1;17(7):e0270740. doi: 10.1371/journal.pone.0270740 (PMC9249166; doi:10.1371/journal.pone.0270740)
Supplement: S2 Table — (DOCX) [file pone.0270740.s002.docx]

**S2 table.** DOR of clinical manifestations for other epidemic weeks, stratified by age group and comorbidities.

|  | **0-4 years** | **5-14 years** | **15-64 years** | **≥65 years** | **Comorbidities** | **No comorbidities** |
| --- | --- | --- | --- | --- | --- | --- |
|  | **DOR**  **(95% CI)** | **DOR**  **(95% CI)** | **DOR**  **(95% CI)** | **DOR**  **(95% CI)** | **DOR**  **(95% CI)** | **DOR**  **(95% CI)** |
| **Case definition** |  |  |  |  |  |  |
| ECDC ELI | 1.21 (0.89-1.63) | 1.17 (0.89-1.54) | 1.43 (1.17-1.76) | 2.29 (1.31-4.00) | 2.44 (1.58-3.75) | 1.26 (1.09-1.46) |
| WHO ILI | 2.12 (1.41-3.20) | 2.10 (1.49-2.95) | 2.22 (1.77-2.78) | 3.55 (1.94-6.48) | 3.33 (1.98-5.60) | 2.22 (1.87-2.63) |
| **Clinical symptoms** |  |  |  |  |  |  |
| Fever | 2.44 (0.84-7.10) | 1.99 (0.87-4.55) | 2.37 (1.70-3.29) | 3.88 (1.98-7.62) | 2.56 (1.33-4.93) | 2.75 (2.07-3.66) |
| Cough | 1.86 (1.21-2.87) | 2.09 (1.47-2.98) | 1.81 (1.40-2.34) | 2.18 (0.89-5.36) | 2.71 (1.36-5.38) | 1.84 (1.53-2.23) |
| Malaise | 1.70 (1.26-2.30) | 1.04 (0.78-1.39) | 1.24 (0.96-1.61) | 1.93 (0.97-3.85) | 1.58 (0.84-2.95) | 1.25 (1.07-1.47) |
| Headache | 2.58 (1.67-3.97) | 1.36 (1.03-1.78) | 1.11 (0.90-1.37) | 1.48 (0.85-2.58) | 1.65 (1.08-2.52) | 1.32 (1.14-1.53) |
| Myalgia | 2.25 (1.41-3.59) | 1.19 (0.89-1.59) | 1.64 (1.25-2.15) | 2.65 (1.38-5.06) | 1.46 (0.94-2.27) | 1.20 (1.04-1.39) |
| Sore throat | 1.46 (1.05-2.04) | 0.72 (0.54-0.95) | 0.87 (0.71-1.07) | 0.92 (0.53-1.60) | 0.93 (0.61-1.43) | 1.03 (0.89-1.19) |
| Shortness of breath | 0.25 (0.10-0.63) | 0.70 (0.33-1.46) | 0.63 (0.40-0.99) | 0.61 (0.29-1.29) | 0.81 (0.47-1.39) | 0.41 (0.27-0.60) |
| Sudden onset of symptoms | 0.97 (0.71-1.33) | 1.12 (0.85-1.47) | 1.31 (1.07-1.62) | 1.88 (1.08-3.28) | 1.99 (1.30-3.06) | 1.13 (0.97-1.31) |

DOR: Diagnostic Odds Ratio; Se: Sensitivity, Sp: specificity
