# Supplementary material for: Does knowing the influenza epidemic threshold has been reached influence the performance of influenza case definitions?
Source: PLoS One. 2022 Jul 1;17(7):e0270740. doi: 10.1371/journal.pone.0270740 (PMC9249166; doi:10.1371/journal.pone.0270740)
Supplement: S3 Table — Influenza sentinel surveillance system, Catalonia, 2008–2018. (DOCX) [file pone.0270740.s003.docx]

**S3 Table.** Sensitivity, specificity, positive predictive value, likelihood ratios, DOR of case definitions and clinical symptoms for the first epidemic weeks and for other epidemic weeks. Influenza sentinel surveillance system, Catalonia, 2008-2018.

|  | **First epidemic weeks** | | | | | | | **Other epidemic weeks** | | | | | |
| --- | --- | --- | --- | --- | --- | --- | --- | --- | --- | --- | --- | --- | --- |
| **Case definition** | **Se (%)** | **Sp (%)** | **PPV (%)** | **Positive LR**  **(95% CI)** | **Negative LR**  **(95% CI)** | **DOR**  **(95% CI)** | **Se (%)** | | **Sp (%)** | **PPV (%)** | **Positive LR**  **(95% CI)** | **Negative LR**  **(95% CI)** | **DOR**  **(95% CI)** |
| ECDC ILI | 64 (58-70) | 45 (38-51) | 52 (46-57) | 1.16 (1.00-1.34) | 0.80 (0.65-1.00) | 1.44 (1.01-2.07) | 57 (55-60) | | 51 (48-53) | 61 (59-63) | 1.17 (1.10-1.24) | 0.84 (0.78-0.89) | 1.40 (1.23-1.59) |
| WHO ILI | 82 (77-87) | 33 (28-40) | 53 (48-58) | 1.23 (1.11-1.37) | 0.54 (0.39-0.74) | 2.30 (1.52-3.50) | 82 (81-84) | | 33 (30-35) | 62 (60-64) | 1.22 (1.17-1.27) | 0.54 (0.48-0.61) | 2.25 (1.94-2.62) |
| Fever | 95 (91-97) | 15 (11-20) | 51 (46-56) | 1.12 (1.06-1.19) | 0.33 (0.17-0.60) | 3.46 (1.77-6.76) | 95 (94-96) | | 13 (11-15) | 60 (58-61) | 1.09 (1.07-1.11) | 0.38 (0.31-0.48) | 2.84 (2.23-3.61) |
| Cough | 87 (82-91) | 20 (15-25) | 50 (45-55) | 1.09 (1.01-1.18) | 0.65 (0.43-0.97) | 1.68 (1.04-2.74) | 87 (85-88) | | 22 (20-24) | 60 (58-62) | 1.10 (1.07-1.14) | 0.62 (0.54-0.72) | 1.77 (1.49-2.10) |
| Malaise | 65 (59-71) | 29 (23-35) | 46 (41-51) | 0.92 (0.82-1.04) | 1.20 (0.93-1.55) | 0.77 (0.53-1.12) | 73 (71-75) | | 31 (29-33) | 59 (57-61) | 1.06 (1.01-1.10) | 0.87 (0.79-0.97) | 1.21 (1.05-1.39) |
| Headache | 50 (44-57) | 64 (58-70) | 57 (50-63) | 1.41 (1.15-1.73) | 0.77 (0.66-0.90) | 1.83 (1.28-2.61) | 51 (49-53) | | 56 (54-59) | 61 (59-63) | 1.17 (1.09-1.25) | 0.87 (0.82-0.93) | 1.34 (1.18-1.52) |
| Myalgia | 54 (48-61) | 48 (41-54) | 49 (43-55) | 1.04 (0.88-1.22) | 0.96 (0.80-1.16) | 1.08 (0.76-1.53) | 57 (55-59) | | 49 (47-52) | 60 (58-62) | 1.12 (1.05-1.19) | 0.88 (0.82-0.94) | 1.27 (1.12-1.45) |
| Sore throat | 50 (44-56) | 43 (37-49) | 45 (39-51) | 0.88 (0.74-1.04) | 1.16 (0.96-1.40) | 0.76 (0.53-1.08) | 51 (49-53) | | 50 (47-52) | 58 (56-60) | 1.02 (0.96-1.09) | 0.98 (0.92-1.04) | 1.05 (0.92-1.19) |
| Shortness of breath | 3 (1-6) | 94 (90-96) | 33 (16-55) | 0.54 (0.24-1.24) | 1.03 (0.99-1.07) | 0.53 (0.22-1.25) | 4 (3-5) | | 92 (91-94) | 40 (33-47) | 0.49 (0.38-0.65) | 1.04 (1.02-1.06) | 0.47 (0.36-0.63) |
| Sudden onset of symptoms | 69 (62-75) | 38 (33-45) | 51 (45-56) | 1.12 (0.98-1.27) | 0.81 (0.64-1.04) | 1.38 (0.95-1.99) | 63 (61-65) | | 43 (40-45) | 60 (58-62) | 1.09 (1.04-1.15) | 0.88 (0.81-0.95) | 1.25 (1.09-1.42) |

DOR: Diagnostic odds ratio; Se: Sensitivity, Sp: specificity, PPV: positive predictive value
