# Supplementary material for: Does knowing the influenza epidemic threshold has been reached influence the performance of influenza case definitions?
Source: PLoS One. 2022 Jul 1;17(7):e0270740. doi: 10.1371/journal.pone.0270740 (PMC9249166; doi:10.1371/journal.pone.0270740)
Supplement: S4 Table — Influenza sentinel surveillance system, Catalonia, 2008–2018. (DOCX) [file pone.0270740.s004.docx]

**S4 Table.** Sensitivity, specificity, positive predictive value, likelihood ratios, DOR of case definitions and clinical symptoms for the first three epidemic weeks and for other epidemic weeks. Influenza sentinel surveillance system, Catalonia, 2008-2018.

|  | **First three epidemic weeks** | | | | | | | **Other epidemic weeks** | | | | | |
| --- | --- | --- | --- | --- | --- | --- | --- | --- | --- | --- | --- | --- | --- |
| **Case definition** | **Se (%)** | **Sp (%)** | **PPV (%)** | **Positive LR**  **(95% CI)** | **Negative LR**  **(95% CI)** | **DOR**  **(95% CI)** | **Se (%)** | | **Sp (%)** | **PPV (%)** | **Positive LR**  **(95% CI)** | **Negative LR**  **(95% CI)** | **DOR**  **(95% CI)** |
| ECDC ILI | 59 (56-62) | 48 (45-52) | 58 (55-62) | 1.15 (1.05-1.25) | 0.85 (0.76-0.94) | 1.35 (1.11-1.65) | 58 (55-60) | | 51 (48-54) | 61 (58-64) | 1.18 (1.09-1.26) | 0.83 (0.77-0.90) | 1.41 (1.22-1.64) |
| WHO ILI | 81 (78-83) | 33 (29-36) | 60 (57-62) | 1.20 (1.13-1.28) | 0.59 (0.49-0.70) | 2.05 (1.63-2.58) | 83 (81-85) | | 33 (30-35) | 62 (60-64) | 1.23 (1.18-1.29) | 0.52 (0.45-0.59) | 2.39 (2.00-2.86) |
| Fever | 95 (93-96) | 15 (12-18) | 58 (55-60) | 1.11 (1.07-1.15) | 0.36 (0.26-0.50) | 3.08 (2.14-4.43) | 95 (94-96) | | 12 (11-14) | 59 (57-61) | 1.09 (1.06-1.11) | 0.39 (0.29-0.50) | 2.82 (2.11-3.76) |
| Cough | 86 (83-88) | 20 (17-23) | 57 (54-59) | 1.07 (1.02-1.12) | 0.72 (0.58-0.90) | 1.48 (1.14-1.93) | 87 (85-89) | | 22 (20-25) | 60 (58-62) | 1.12 (1.08-1.16) | 0.58 (0.49-0.68) | 1.93 (1.58-2.36) |
| Malaise | 69 (66-72) | 29 (26-33) | 54 (51-57) | 0.97 (0.91-1.04) | 1.06 (0.91-1.23) | 0.92 (0.74-1.14) | 74 (72-76) | | 32 (29-34) | 59 (57-61) | 1.08 (1.03-1.13) | 0.83 (0.73-0.93) | 1.31 (1.11-1.54) |
| Headache | 51 (47-54) | 58 (55-62) | 60 (56-63) | 1.22 (1.09-1.36) | 0.84 (0.77-0.93) | 1.44 (1.18-1.76) | 51 (48-53) | | 57 (54-60) | 61 (58-64) | 1.18 (1.06-1.28) | 0.86 (0.80-0.93) | 1.37 (1.18-1.59) |
| Myalgia | 57 (54-60) | 50 (46-53) | 58 (55-61) | 1.13 (1.03-1.24) | 0.87 (0.78-0.97) | 1.30 (1.07-1.59) | 56 (54-59) | | 49 (46-52) | 59 (57-62) | 1.10 (1.02-1.18) | 0.90 (0.83-0.98) | 1.22 (1.05-1.42) |
| Sore throat | 53 (50-57) | 47 (44-51) | 55 (52-59) | 1.01 (0.92-1.11) | 0.99 (0.89-1.10) | 1.02 (0.84-1.24) | 50 (48-53) | | 50 (47-53) | 57 (54-60) | 1.00 (0.93-1.08) | 1.00 (0.93-1.08) | 1.00 (0.86-1.16) |
| Shortness of breath | 3 (2-5) | 92 (90-94) | 36 (26-47) | 0.45 (0.29-0.70) | 1.05 (1.02-1.07) | 0.43 (0.27-0.68) | 4 (3-5) | | 93 (91-94) | 41 (33-50) | 0.53 (0.38-0.73) | 1.04 (1.02-1.06) | 0.51 (0.36-0.72) |
| Sudden onset of symptoms | 64 (61-68) | 41 (37-45) | 57 (54-60) | 1.09 (1.00-1.18) | 0.87 (0.77-0.99) | 1.24 (1.01-1.53) | 63 (60-65) | | 43 (40-46) | 59 (57-62) | 1.10 (1.03-1.17) | 0.87 (0.80-0.96) | 1.26 (1.08-1.46) |

DOR: Diagnostic odds ratio; Se: Sensitivity, Sp: specificity, PPV: positive predictive value
